# Editorial: PROTACs: Targeted therapies for cancer treatment

**DOI:** 10.3389/fcell.2023.1102721

**Published:** 2023-02-03

**Authors:** Sobia Tabassum

**Affiliations:** Department of Biological Sciences, International Islamic University, Islamabad, Pakistan

**Keywords:** Research Topic, proteolysis-targeting chimeric molecule (PROTAC), cancer treatment, state of art, cutting edge technology

## Introduction

Cancer is one of the leading cause of morbidity and mortality worldwide. The development of chemotherapeutic drug resistance, which causes the disease to reoccur, has decreased the effectiveness of cancer treatments. Proteolysis-targeting chimaera (PROTACs) are protein targeting chimeric molecules that have the capacity to bind disease-causing protein of interest with a high degree of specificity and then lyse it. PROTACs are specialized, adaptable, and biologically active. They have tremendous advantages over other types of inhibitors which are not able to give protection against the drug resistivity Research Topic in cancer as they have the ability to trigger selective intracellular proteolysis, which can be utilized to specifically target and destroy tumor-promoting proteins inside of cells. To enhance their therapeutic outcomes, new understandings of the molecular mechanisms of PROTAC-mediated degradation are required.

## Aim and objectives

The current Research Topic aims to explore the most recent developments in the creation and use of PROTACS-based treatments for cancer. We invite research articles, review articles, and viewpoints that address to the following Research Topic, but are not restricted to them.

• Molecular underpinnings of PROTAC-mediated protein degradation
• Modulation of the ubiquitination system for cancer treatment
• Innovative designing and development of PROTACS-based therapeutics
• Comparison of PROTACs with non-targeted and targeted therapies

This Research Topic brings together several scientific contributions that highlight some extremely exciting findings. The published articles on this Research Topic have attracted significant interest from both academia and industry.

Zheng et al. submitted a comprehensive review on this subject in order to address cancer drug resistance, which is a significant obstacle to the successful treatment of malignancies going forward. Recent developments in this field of study show that PROTACs can successfully destroy targets that confer cancer resistance to therapies, building the foundation for next-generation therapies and that provide additional clinical benefit to patients.

The authors provided an overview of the current use of PROTACs to treat solid tumors and included recommendations for overcoming their clinical development roadblocks. Among designing and development constraints, improvements in bioavailability and safety brought about by an improved delivery route seem to be relevant. In light of this, Juan et al. have proposed methods to enhance their therapeutic efficacy and the development of fine-tuned nanoparticles based delivery systems to increase the bioactivity of PROTACs.

Significant developments in newly developed degrader technologies were also reported in this area (Luo et al.). The emphasis of the authors has been on the molecular design of PROTACs using various methodologies, which provides a deeper mechanistic knowledge of developing degraders and serves as a beneficial roadmap for the advancement of the upcoming degrader technologies. Anwar et al. published a very thorough analysis of PROTACs designed to combat different forms of leukaemia and blood cancers. Along with all of this material, another investigation focused on how YAP1 deacetylation promoted chemotherapy resistance and targeted treatment in FLT3-ITD^+^ AML cells (Feng et al.). This research demonstrates that the FLT3-ITD^+^ AML cells are maintained by the HDAC10-YAP1-PARP1 axis, and blocking this axis may improve the clinical outcomes for FLT3-ITD^+^ AML patients.

We anticipate that this Research Topic will offer an informative guide to understanding this cutting-edge technology which can be employed as tools for targeted therapies and drug development.
